# Long-Term Recall Error in Retrospective Family Surveys: Cohabitation Histories in Denmark

**DOI:** 10.1007/s10680-026-09775-9

**Published:** 2026-05-29

**Authors:** Peter Fallesen, Lisbeth Trille G. Loft, Emil A.L. Simonsen, Jolien Cremers, Laust Hvas Mortensen

**Affiliations:** 1 Stockholm University, Stockholm, Sweden; 2 ROCKWOOL Foundation, Copenhagen, Denmark; 3 University of Copenhagen, Copenhagen, Denmark; 4 Statistics Denmark, Copenhagen, Denmark

**Keywords:** Cohabitation, Generation and gender survey, Measurement error, Recall error, Survey design

## Abstract

Recall questions provide the possibility of obtaining longitudinal information in cross-sectional surveys and are often used in family research. Yet, recall instruments may suffer from measurement issues. This study links a recent cross-sectional survey that includes recall histories of all unions involving cohabitation to administrative cohabitation records for Denmark and assesses the recall error in cohabitation status histories. Under mild assumptions, it distinguishes recall error from other survey error types, allowing for assessing the increase in recall error as time to the recalled period increases. The quality of recall for living with a partner decreases in a linear fashion by 0.5–0.6% points per year on average over a 20-year period. Results are robust to a series of sensitivity tests. The presented estimate of recall error for cohabitation status likely provides a lower bound for general recall error and shows by example how regression calibration methods can be used to correct for recall error even if only a small random subset of data includes true values. Recall instruments used in family research are prone to recall error, and failing to account for this may lead to biased estimates especially for longer recall periods.

**Supplementary Information:**

The online version contains supplementary material available at 10.1007/s10680-026-09775-9.

## Introduction

Family demographers studying the life course often rely on survey data that includes information recalled over a lengthy period of time, where respondents are asked to recall key events such as family and fertility histories as these evolved across the life course (e.g., Andersson et al., 2017; Hayford & Morgan, 2008; Kreyenfeld et al., 2013; Kreyenfeld, 2013; Perelli-Harris & Lyons-Amos, 2015). Across social sciences, other usage includes health histories (Ayhan & Işiksal, 2004; Haas et al., 2011; Johnson & Schoeni, 2011), food intake and security histories (Gertler et al., 2017; Gibson & Kim, 2007; Wollburg et al., 2021), labor market and educational histories (Bingley & Martinello, 2017; Jürges, 2007; Manzoni et al., 2010), childhood circumstances (Berney & Blane, 1997; Francesconi, 2005; Havari & Mazzonna, 2015), and migration histories (Auriat, 1991). Recalled information in cross-sectional surveys allows researchers to capture longitudinal data in a cheaper way that requires less long-term investment and planning than a longitudinal survey (see Powers et al., 1978 for an early discussion). However, the recalled information may suffer from error (Beckett et al., 2001; Dalziel et al., 2018; Kjellsson et al., 2014; Kreyenfeld et al., 2013; Kreyenfeld, 2013; Ruckdeschel et al., 2016) leading to measurement issues that may bias results (Gibson & Kim, 2010; Manzoni et al., 2010). Further, the recall error may also increase with length of the recall period (Kjellsson et al., 2014).

Previous work examining the quality of recall across multiple surveys have cautioned researchers that long-term recall is likely error-prone (Hayford & Morgan, 2008), but to date several types of recalled information, including cohabitation histories, are yet to be quantified against a ’gold standard’ long-term baseline. Understanding the magnitude of recall error associated with different areas of life histories, and how it evolves the further back information is recalled, will allow researchers to better account for the bias recall error may introduce to analyses, especially when it increases with time.

In this study, we consider recall error in cohabitation histories using the recent Danish wave of the Generation and Gender Survey (Simonsen et al., 2021) in combination with updated high-quality administrative housing registers supplied and maintained by Statistics Denmark. In essence, this study is a reverse record check conducted over a 20-year period. Under the assumption that people fully know if they are cohabiting with a partner at time of answering the survey, we first establish the level of match error between survey data and administrative records. Our baseline level of identification of cohabiting couples is in the same range as recent efforts to identify married couples in Germany using a similar strategy (Goldschmidt et al., 2017). We use the identified measure of baseline match error to evaluate to what extent error increases when respondents are asked to recall cohabitation histories back in time, essentially approaching recall error as a form of classification error. Our results suggest a baseline match error for living with a partner of 8% and that recall error increases consistently with an average of 0.5–0.6% point for each year back in time that respondents were required to recall. This finding is robust to a series of sensitivity analyses and is in accordance with a situation where respondents misreport rather than misremember their timing.

This study contributes important new information on sources of measurement error under recall in general, and for cohabitation histories in particular. Most previous studies of recall error have either had to triangulate between surveys (e.g., Beckett et al., 2001; Hayford & Morgan, 2008; Manzoni et al., 2010), rely on shorter period of recall (e.g., Ayhan & Işiksal, 2004; Dalziel et al., 2018; Kjellsson et al., 2014), compared consistency across survey waves in historical recall (Havari & Mazzonna, 2015), compared very small samples to official records (e.g., Auriat, 1991), or for long-term recall focused on older individuals recalling far back events (Berney & Blane, 1997; Haas et al., 2011; Havari & Mazzonna, 2015; Johnson & Schoeni, 2011). Applying only mild assumptions, this study examines how recall error develops against a clearly defined baseline as the length of the period recalled increases across two decades among a sample of younger individuals (18–49 years of age). Given the high application rate of recall questions of cohabitation in surveys such as the National Survey of Family Growth in the US, the Generation and Gender Survey that covers more than 20 countries at time of writing, and the World Fertility Survey, the knowledge put forward here allow researchers to engage with bias arising from recall error in a more informed way, including the possibility of quantitatively modelling different parameters of interest to incorporate recall error. Further, we also show that the estimated recall error develops at a steady rate across the time horizon considered in the study, thereby allowing future studies to make more informed assumptions about longer term error development than previously available in the literature. Last, we demonstrate that if survey collectors are able to verify key recall variables from ‘objective’ sources for a small, randomly chosen subset of the data, recall error can easily be addressed with calibration methods. Such ‘gold standard’ measures may of course not be available for all outcomes (see however Auriat, 1991; Bingley & Martinello, 2017 for examples of comparisons to “objective” sources), and when available have with their own measurement error issues. In the Conclusion section, we present several examples of scenarios where obtaining objective data for calibration could be feasible.

The study examines a salient and objectively defined event in the life course: Cohabitation. The literature has previously found that recall quality is higher for more salient events (Beckett et al., 2001), compared to more subjective or context-specific life course events [e.g., perceived economic hardship during childhood (Jivraj et al., 2020) or illegal substance use (Beckett et al., 2001)]. Further, the sample consists of relatively young individuals (age 18–49), who should not be facing any form of age-related cognitive decline. For this reason, we suggest that the rate of development for recall error reported in the present study should be considered a lower bound both in terms of the saliency of the event recalled, and the age of those recalling it.

## Background

### Recall Error in Survey-Based Research

The use of recall questions as survey-instruments to obtain longitudinal information has a long history. Within family research, for example, recall instruments allow researchers access to the whole of a respondent’s relationship and fertility histories through one round of data collection (e.g., Beckett et al., 2001). Alternatively, longitudinal data collections are needed, which are costly, prone to attrition issues, and require researchers to invest substantial time (sometimes most of a career) before the family histories become of a meaningful length for life course analysis. Still, recall instruments do present issues of their own. Powers et al. (1978) provide an early discussion of the pitfalls of using recall instruments instead of investing in longitudinal data collection, highlighting the potential for recall error. One key example is recall error with regard to cohabitation histories, which may occur for several reasons. First, respondents may simply fail to retrieve the cohabitation when answering the instrument (recall loss). Second, respondents may reconstruct the history incorrectly, such as to misremember the timing (telescoping). Third, respondents may actively choose not to report the event out of survey-fatigue, or because they do not want to recall an unpleasant or inappropriate relationship (avoidance). Whereas the recall loss and telescoping represent misremembering, survey-fatigue and avoidance are best described as misreporting. Regardless of type, they all contribute to recall error insofar as their magnitude increase with length of time recalled.

### A Statistical Framework for Recall Error

The volume of recall error likely increases as people recall further back in time (as suggested in related settings by, e.g., Beckett et al., 2001; Hayford & Morgan, 2008; Kjellsson et al., 2014; Macdermid, 1989; Reimer, 2004) leading to a non-random (or ‘non-classical’) measurement error for which conventional statistical corrections are less effective (Gibson & Kim, 2010). Specifically, this type of measurement error occurs when the error in measuring the variable of interest is correlated with the true value of that variable, with the true value of other variables in the model, or with the errors in measuring those other variables (Bound et al., 2001). Thus, when using information collected by survey-based recall instruments, it is important to try to ascertain both the magnitude, nature, and source of potential recall error. In this study, we show by example of cohabitation status, (a) the quantification of the magnitude of recall error in a binary variable setting, (b) how recall error evolves the further back in time the recall runs, and (c) examine potential sources of the identified recall error by means of sensitivity analyses.

A convenient statistical framework to model recall error is the linear measurement error model:

1 $$\:\begin{array}{c}{X}^{*}={a}_{0}+{a}_{x}X+U\:\end{array}$$

This model encompasses a situation where the observed measurement $\:{X}^{*}\:$includes both random error $\:U$ with mean 0 and independent of the true value $\:X$ and systematic error described by $\:{a}_{0}$ and $\:{a}_{x}$ that is dependent on $\:X$ and possibly other variables. In Eq. (1), $\:{a}_{0}$ quantifies location bias and $\:{a}_{x}$ quantifies the scale bias which is bias that depends on the value of $\:X$. Various extensions of the linear measurement error model include specifying $\:{a}_{0}$ as a random variable varying across individuals, allowing $\:{X}^{*}$ to depend on other variables $\:\stackrel{\sim}{Z}$ or allow the variance of $\:U$ to depend on $\:X$.

In this paper we quantify and adjust for the recall error in cohabitation status, a binary categorical variable, which we assume is to be used as a covariate in a linear regression model. For (binary) categorical variables measurement error is also referred to as misclassification and Eq. (1) is specified as a logistic regression model:

2 $$\:\begin{array}{c}P\left({X}^{*\:}=1\right)={logit}^{-1}\left({a}_{0}+{a}_{x}X\right).\end{array}$$

The bias in estimates of regression coefficients caused by error in binary variables depends on whether it is used as an outcome or covariate, as well as whether we assume non-differential measurement error. That is, whether it is independent of the outcome $\:Y$ and covariates $\:Z$ in a regression model, or equivalently that they are independent of the random errors of the regression model of interest. Keogh et al. (2020) give an excellent overview of how measurement error biases inference in regression models. In short, for our case of a binary covariate in a linear regression model, under the assumption of non-differential error and when used as a covariate, the regression coefficient is biased. Still, testing the hypothesis $\:{\beta\:}_{x}^{*}=0$ is a valid test of $\:{\beta\:}_{x}=0$ although it has somewhat less power.

## Methods

### Data

To empirically assess recall error by example of cohabitation status, we combined two independent data sources: (1) The Generation and Gender Survey (GGS) 2020 for Denmark which was collected between March 15th and June 10th, 2021, and (2) the Danish Central Population Register (CPR) maintained by Statistics Denmark. The GGS included 7232 complete responses from a random gross sample of men and women aged 18–49 living in Denmark (Simonsen et al., 2021). The gross sample was drawn by Statistics Denmark from the full Danish population within gender (men were oversampled to account for lower response rates for that gender), and thus reflect a representative sample of the underlying population within gender. Respondents were asked to first record if they were currently living with a partner including the month and year the present cohabitation had begun. The respondents were then asked to record *all* previous cohabiting relationships (married or unmarried) from year and month of start of the cohabitation to the year and month of end of the cohabitation. It was possible for Statistics Denmark to link all GGS participants to CPR data by means of a unique personal identification number that all Danish residents are assigned at birth or date of immigration, and which was used for the initial sampling for the GGS. The Danish CPR data provides information on residency down to the apartment level, allowing us to establish whether a respondent is cohabiting according to these administrative data as of December 31 st of every year.

### Measures

#### Cohabitation Status

In the GGS-data cohabitation status was recorded as living with a partner (married or unmarried), and in the CPR-records a cohabiting union is defined as sharing the same address and either being (a) married to each other; (b) having a child together; or (c) being the sole two adults in the household, of opposite sex, not directly related by blood, and within 15 years of age of each other. In this study cohabitation status was measured as a binary variable indicating whether a respondent was living with a partner (married or unmarried) or not living with a partner, and thus the variable in the CPR-records were recoded as such. In the GGS-data respondents were asked to report the month and year of when living with a given partner began and ended, and similarly in the CPR-records it is possible to obtain day, month and year for the beginning and end of any given cohabitation. However, to condense the here presented results, this study uses only annual data comparing a respondents’ reported cohabitation status in the GGS-survey with that of the CPR-records. Specifically, we compare the match of respondents’ cohabitation status in the two data sources on January 1 st each year from 2001 to 2021, with 2021 serving as the baseline year.

#### Defining Recall Error

This study assumes that respondents knew whether they were cohabiting on the day they completed the survey. If so, any discrepancy between CPR records and survey responses for that day cannot reflect recall error. It can only reflect baseline match error between the two data sources plus survey response error. This allows us to decompose recall error for a year, t, into two separate components:

3 $$\:\mathrm{Recall\:}{\mathrm{error}}_{t}\mathrm{\:=\:}\mathrm{Match\:}{\mathrm{error}}_{t}-\mathrm{Baseline\:match\:error}$$

The baseline match error is the share of mismatch between respondents’ cohabitation status in the GGS and their cohabitation status in the 2021 CPR records. To get the baseline match error we compare respondents’ cohabitation status in the GGS with their status in the CPR records. Baseline match error could originate either in the GGS-data or in the CPR-records. In the GGS-data, this could be due to (1) respondents filling out the survey-instrument wrong or (2) non-response. In the CPR-records it can stem from four sources, namely (1) two people of opposite gender living together but not in a relationship (co-living), (2) being a part of a multifamily household, (3) unmarried same-sex couple; and (4) unregistered move or erroneous registration. Importantly, we assume that none of these are related to recall error beyond through observable characteristics.

Match error in year *t* is the difference between cohabitation status in the GGS in the year *t* (prior to 2021) and the CPR records for the same year. As the CPR records are measured the same way across all years included in the study period, we assume that the baseline level of mismatch remains constant. Thereby, any difference between the baseline match error and the match error for year *t* would then be recall error. For example, if 8% of records mismatch at baseline (2021) and 14% mismatch when recalling 2011, the recall error for 10-year recall is 14% − 8% = 6%. We can further separate the recall error by cohabitation status, by using the cohabitation status recorded in the CPR as denominator. That is, assuming that CPR is the ‘objective’ measure, does recall error differ by status in the CPR records.

#### Predictors of Match Error

In addition to cohabitation status (living with a partner or not) as recorded in the CPR, a set of predictors measured was included to further investigate heterogeneity in the identified recall error. These predictors were gender (being female), parenthood (having at least one child), and age (grouped into six categories of age 45–49, 40–44, 35–39, 30–34, 25–29 and 18–24).

### Analytical Strategy

#### Estimating Recall Error

Recall error was in this study defined as the difference in the baseline match error and the error of any given recalled year. In estimating the recall error, a second assumption is introduced to the study, namely that the rate of match error is constant over time. Applying this assumption, the difference between the baseline match error and the error of given year recalled denotes the recall error. The empirical application of this approach is to regress (a) the baseline match error (year 2021) and (b) the match error at any given recall year (year 2001 through 2020) on the cohabitation status from the CPR-records. Generally, this can be written as:

4 $$\:{Y}_{ir}={X}_{ir}\gamma\:+{Cohab}_{ir}\delta\:+\:{\upepsilon}_{ir}\:,\:i=\:\left\{1,2\right\}$$

and in the specific case of this study, the estimation procedure is defined as:

5 $$\begin{gathered} {Y}_{1r}^{2021}=\:{\beta\:}_{0}^{2021}+{\boldsymbol{B}\boldsymbol{Y}}_{\boldsymbol{r}\:}\:{\boldsymbol{\gamma\:}}_{}^{2021}+\:{\delta\:}_{}^{2021}\:{Cohab}_{r}^{2021}+\:{\upepsilon}_{r}^{2021} \hfill \\ {Y}_{2r}^{t}=\:{\beta\:}_{0}^{\mathrm{t}}+{\boldsymbol{B}\boldsymbol{Y}}_{\boldsymbol{r}\:}\:{\boldsymbol{\gamma\:}}_{}^{\mathbf{t}}+\:{\delta\:}_{}^{\mathrm{t}}\:{Cohab}_{r}^{t}+\:{\upepsilon}_{r}^{t}\end{gathered} $$

where $\:{Y}_{1r}^{2021}$ is the indicator of cohabitation status being identical in the CPR-records and the GGS-data in year 2021, *BY* is a vector of birth years, $\:{Cohab}_{1r}^{2021}$ in an indicator of cohabitation status equal to living with a partner in the CPR-records in 2021, *X* is the vector of control variables and $\:{\upepsilon}_{1r}$ is the error term where $\:E\left[\:{\upepsilon}_{1r}|X\right]={\sigma\:}_{1}$. Likewise, $\:{Y}_{2r}^{t}$ is an indicator of cohabitation status being identically recorded in the CPR-records and the GGS-data in recall year *t*, *BY* is a vector of birth years, $\:{Cohab}_{2r}^{t}$ in an indicator of cohabitation status equal to living with a partner in the CPR-records in recall year *t*, and $\:{\upepsilon}_{2r}$ is the error term where $\:E\left[\:{\upepsilon}_{2r}|X\right]={\sigma\:}_{2}$. Separate estimates are provided depending on the cohabitation status (living with a partner or not) as recorded in the CPR-records, and the obtained cohabitation-specific means are adjusted accordingly:

6 $$\:\mathrm{R}\mathrm{e}\mathrm{c}\mathrm{a}\mathrm{l}\mathrm{l}\:\mathrm{e}\mathrm{r}\mathrm{r}\mathrm{o}\mathrm{r}\:\mathrm{i}\mathrm{n}\:t=(\widehat{{Y}_{2r}^{\mathrm{t}}}\left|\:Coha{b}_{2r}^{\mathrm{t}}=c,\mathrm{B}\mathrm{Y})-(\widehat{{Y}_{1r}^{2021}}\:\right|\:Coha{b}_{1r}^{2021}=c,BY)$$

Note that the recall error for year *t* can be interpreted as the proportion of the sample for which the observed measurement $\:{X}^{*}\:$is not equal to the true value $\:X\:$. Thus, it can thereby be related to the logistic measurement error model from Eq. (2). This correspondence is further used in the provided practical example for adjusting for recall error.

In sum, this study assesses if recall estimates differ from baseline, treating recall error as a prediction error composite of baseline match error and recall, with $\:1-{Y}_{1r}^{2021}$ serving as the prediction (or, match) error under no recall error. To obtain correct standard errors for the estimated recall error, we use a seemingly unrelated regression to estimate Eq. (6) separately by each year *t*, and then compute the standard error for the recall error by relationship status as:

7 $$\:{{\upsigma\:}}_{\mathrm{R}\mathrm{E}}^{}=\sqrt{{{\upsigma\:}}_{2021}^{2}+{\sigma\:}_{t}^{2}-2{\sigma\:}_{2021}{\sigma\:}_{t}}$$

where $\:{\sigma\:}_{2021}$ is the standard error of the prediction of the baseline match in 2021 and $\:{\sigma\:}_{t}$ is the standard error of the prediction for the match at recalled year *t*.

#### Sensitivity Analyses

To examine the robustness of our results, we performed a series of five sensitivity tests. First, we allowed respondents’ recall to be off by one year more or less. For example, if a respondent recalled a spell of living with a partner in 2005 in the GGS-data, any record of that respondent living with a partner in 2004, 2005 or 2006 in the CPR-records was viewed as a match (one-year margin). Second, to assess if the identified recall error was an artefact of younger cohorts aging out of the window of observation (age 18–49 between 2001 and 2021) as the recall period increased, we limited the sample to only those above age 18 in Denmark in year 2000 (in sample 2000). Third, to investigate if recall error was driven by respondents becoming younger as the recall period increases (and younger individuals likely having different living arrangements), we predicted the baseline match error at the respondents age at the time of the given recall year instead of the age at GGS participation (baseline by age in recall year). Fourth, as respondents may not want to provide their cohabitation histories in the GGS-data (the instrument had an option to refuse), we excluded this group from the analysis (excluding refusers). Fifth, we allowed for optimal prediction by fully saturating both estimated equations by means of an interaction term for cohabitation status and birth year.

#### Additional Adjustments

Unless specified, all results are weighted to the age and gender composition of the full Danish female population aged 18–49. In addition, for each recalled year, we excluded those who either were not in the country or who were below 18 that year. Using weighted data allows for a more informative interpretation of the baseline match error (i.e., what is the quality of the administrative cohabitation information for the age group 18–49 in Denmark). Unweighted data produce quantitatively similar results to the weighted data for the main analysis and can be found in Appendix.

## Results

### Descriptive Findings

#### Sample Characteristics

Although the GGS-data were sampled to closely mirror the Danish population age 18–49, some discrepancies between the final GGS sample and the population did occur. Because of the high quality of the Danish CPR-records, it is possible to generate rather precise population weights and apply these in our analyses. Table 1 presents the weighted and the unweighted GGS sample characteristics matched with CPR-records. According to the CPR-records and in the weighted sample, about half of the GGS participants (52.07%) were living with a partner at baseline (start of year 2021). This figure is somewhat higher if the sample is not weighted (57.73). Similarly, the initial GGS sample had some issue with females being overrepresented (49.33 vs. 56.04%). Still, taken as a whole, the discrepancies between the population counts and the GGS sample for the variables included in this study were minor. Within the weighted sample, 44.50% had at least one child, and the age groups were relatively evenly distributed with the share of respondents aged 45–49 being slightly larger (19.63%) and share of respondents aged 40–44 being slightly smaller (13.54%).

**Table 1:** Sample characteristics for GGS-data matched with CPR-records *N* = 7273

	Weighted sample (%)	Unweighted sample (%)
Living w. partner at baseline (2021) in CPR-records	52.07	57.73
Gender: female	49.33	56.04
Parenthood: has at least one child	44.50	47.37
Age group in 2021:		
45–49	19.63	18.89
40–44	13.54	15.87
35–39	15.00	15.04
30–34	15.81	13.52
25–29	15.97	14.67
18–24	20.05	22.01

#### Baseline Match Error

Some baseline match error for cohabitation status is identified. Table 2 presents the shares in accordance and in divergence between the GGS-data when matched to CPR-records. There is accordance between the GGS-data and the CPR-records in 92.03% of the cases, yielding a divergence of 7.97%. The match error was 9.03% for those living with a partner according to the CPR data, and 6.74% for those single according to the CPR. Given the definition of cohabiting from the CPR, housemates may cause problems with the classification in two ways. First, two people sharing a dwelling as housemates may be erroneously coded as a couple if they are of opposite sex, unrelated, and within 15 years of each other. Second, unmarried childless couples who share their house with other adults may erroneously be coded as not being in a couple. From the bottom of Table 2, it can be seen that although misclassification due to housemates occurs, there is baseline error left caused either by misreporting in the GGS or in the CPR data.

**Table 2:** Baseline match error (year 2021) for cohabitation status

	Accordance btw. GGS and CPR (%)	Divergence btw. GGS and CPR (%)	Divergence, but reports other people in home in GGS (%)
Overall	92.03	7.97	
Living w. partner in CPR	90.97	9.03	
Not living w. partner in CPR	93.26	6.74	
Living w. partner in CPR	90.97	7.47	1.56
Not living w. partner in CPR	93.26	5.03	1.72

GGS-data matched with CPR-records *N* = 7273, weighted sample

### Recall Error Relative to Baseline Across Number of Recalled Years

**Fig. 1:**
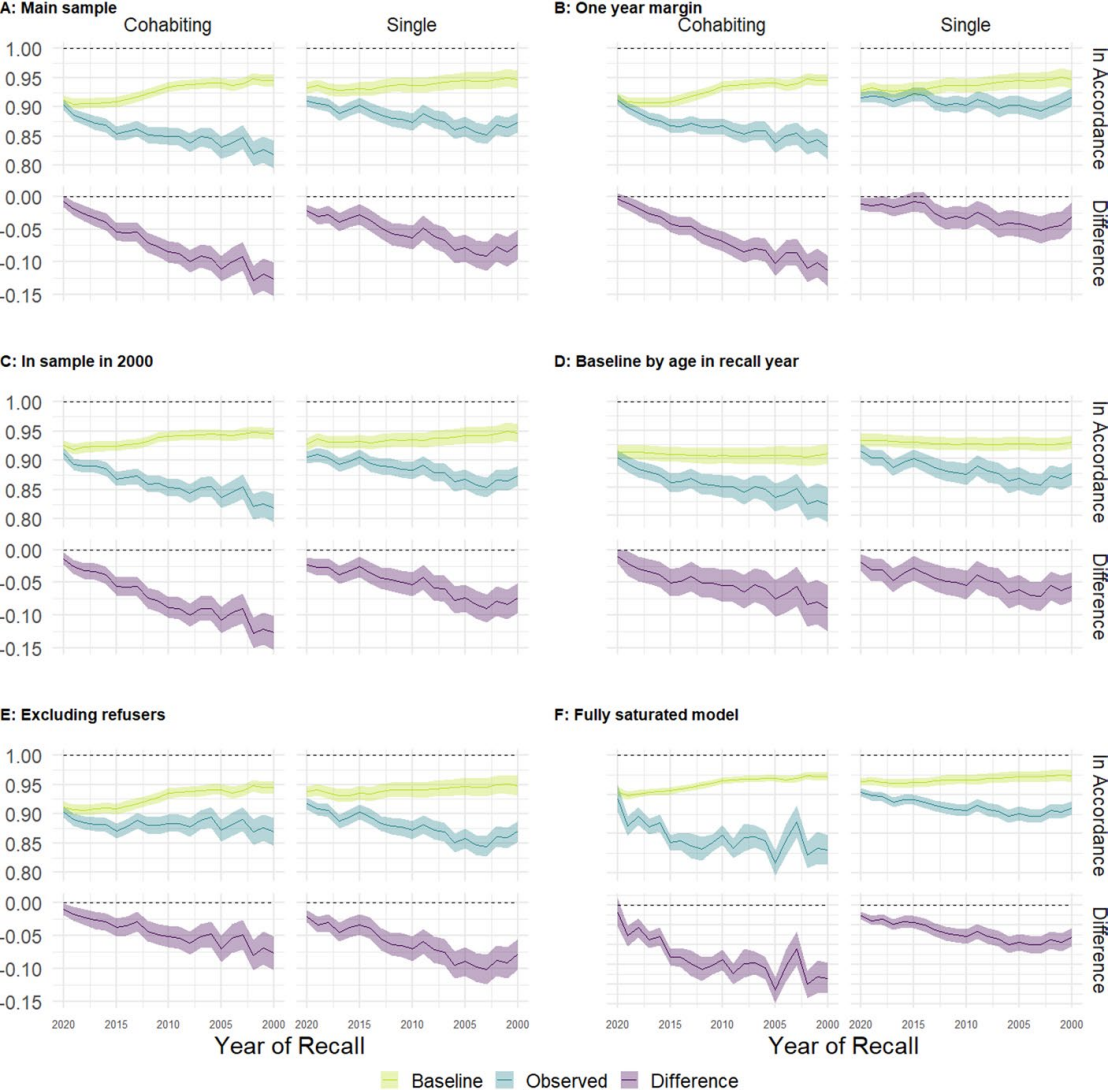
Observed mismatch error compared to baseline mismatch across number of recalled years, by cohabitation status. Source: GGS DK 2020 with population weights and own calculations on data from Statistics Denmark. Standard errors for panel D obtained from 1000 bootstrap replications. Figure 2 in Appendix shows results from unweighted data

Next, we present evidence on the development of recall error over years recalled from our main specification and five different sensitivity tests. Figure 1 presents the results. Recall error (purple band designated “Difference”) expresses the difference between the estimated baseline match error (yellow band designated “Baseline”) and observed match error in the period recalled (blue line designated “Observed”). Plot A in the upper left corner presents results from the main specification, and the remaining five plot reports results from the sensitivity analysis. Across all plots, we see that recall error.

So, how does recall error evolve as the distance between now and the period recalled increases? Table 3 reports the average annual decline in correct classification of cohabiting status over the study period using the main specification from Fig. 1a. Assuming linearity across the time horizon studied, recall error increases with 0.6% for recalling cohabitation (and 0.5% for recalling singlehood) per year relative to 2021 baseline, meaning that at 20 years of recall, recall error has caused the mismatch to increase by 12% for recalling cohabitation and 10% for recalling singlehood. However, when excluding those who did not provide any answer (refusers) the rate declines to 0.5% for both states.

**Table 3:** Average decline in correct classification of cohabitation status per year

	Living with partner	Not living with partner
Annual average decline from baseline	− 0.006	− 0.005
Marginal bias relative to baseline	− 0.6%	− 0.5%

### Adjusting for Recall Error

In the previous section we have modelled the magnitude of the recall error. In this section we demonstrate how to adjust for the bias associated with recall error that follows a linear measurement error model, in a regression model with a continuous outcome, such as income. For the purpose of this example, we add measurement error to a simulated unbiased original sample, and then proceed to show how the correct unbiased estimates can be recovered using regression calibration methods from the mecor R-package (Nab et al., 2021).

*Data.* We have constructed a simulated data dataset that resembles the original GGS data used in the above analyses (available in supplementary materials and labeled simulated_data.csv). Data simulation is implemented in three R-scripts (also available as supplemental materials and labeled GGS_simulated.R, data_simulation.R, and error_correction.R).

The included simulated dataset contains the following variables: ID (id-record for the simulated individual), year (year of observation), sex (sex of the simulated individual), age (age of the simulated individual in the observation year), age (categorical age variable), cohab (simulated cohabitation status with recall error in the observation year, cohab_true (simulated true cohabitation status in the observation year), parent (parental status), outcome (a continuous outcome variable simulated according to the following linear regression model: $\:outcome=\alpha\:+\:{\beta\:}_{sex}*sex+$ $\:{\beta\:}_{age}*age+\:{\beta\:}_{cohab}*coha{b}_{true}+$ $N\left(\mathrm{0,1}\right)=50+0.1*sex+0.2*age+$ $0.5*coha{b}_{true}+N\left[\mathrm{0,1}\right]$).

Note that the data is simulated such that the GGS cohabitation status in 2021 is regarded as the ‘true’ cohabitation status for that year and that an increasing amount of recall error is added at random for the years 2020 − 2000 to create a simulated cohabitation status with error.

In the simulated data a 0.06% recall error for living with a partner is added for each year we go back in time. Because our results on quantifying recall error shows there is a certain amount of baseline match error between the GGS survey data and the CPR data, the simulated true cohabitation status is regarded as the CPR data (‘gold standard’) only for the years 2000–2020. For 2021 no error in cohabitation status (cohab = cohab_true) is simulated since we assume the GGS data is measured without error in this year.

In a real world setting both the GGS data and CPR data contain additional error apart from recall error. For the sake of simplicity in this example we will assume that such ‘random measurement error’ does not induce critical bias in the estimation of regression coefficients.

*Method.* The first step is to adjust for bias induced by recall error in the estimate of the regression coefficient when regressing sex, age, and cohabitation status on the simulated outcome variable (labeled outcome). Five different regression models are fitted to the simulated data (see the R-script: error_correction.R in supplemental materials), (a) one regression model in which the measured cohabitation status is used, (b) one regression model in which the true cohabitation status is used, and (c) three regression calibration models in which information for a random subset of the data on the true cohabitation status is included, and used as a validation set to correct for the recall error in the measured cohabitation status (random subsamples of 75%, 25%, and 5% of the full dataset). Note that these percentages are conditional on the sample size, but do not need to increase with sample size. That is, had our sample been twice as large, we would not need to double the size of the calibration sample (Fox et al., 2020).

To fit the regression calibration models the R-package “mecor” (Nab et al., 2021) is employed. The regression calibration methods from this package rely on the assumption that there is measurement error in one variable at a time (cohabitation status) and thus, that the remaining variables in the model (outcome, sex and age) are measured without error. The methods further assume that the covariate measurement error is non-differential (e.g., errors in the covariate are independent of the random errors in the linear regression model). Finally, the methods assume that the subset of the data for which a validation set is available is a random subset.

Table 4 presents the estimated regression coefficients and standard errors for cohabitation status for each of the five fitted regressions models on the basis of the simulated data. The table reports that the true value of the regression coefficient is 0.5. Using validation sets leads to estimates with no or minimal bias, also when the validation set only contains 25% of observations. Depending on length of recall, no or minimal bias is also observed (for the most recent ten years of recall) even when only using a 5% validation set.

**Table 4:** Estimated regression coefficients for cohabitation status and its standard error according to five regression models (*N* = 7273, N*T = 108,346)

Year	True cohabitation status		Measured cohabitation status		Validation, 75% of observations		Validation, 25% of observations		Validation, 5% of observations	
	$\:{\widehat{{\upbeta\:}}}_{\mathrm{c}\mathrm{o}\mathrm{h}\mathrm{a}\mathrm{b}}$	se($\:{\widehat{{\upbeta\:}}}_{\mathrm{c}\mathrm{o}\mathrm{h}\mathrm{a}\mathrm{b}})$	$\:{\widehat{{\upbeta\:}}}_{\mathrm{c}\mathrm{o}\mathrm{h}\mathrm{a}\mathrm{b}}$	se($\:{\widehat{{\upbeta\:}}}_{\mathrm{c}\mathrm{o}\mathrm{h}\mathrm{a}\mathrm{b}})$	$\:{\widehat{{\upbeta\:}}}_{\mathrm{c}\mathrm{o}\mathrm{h}\mathrm{a}\mathrm{b}}$	se($\:{\widehat{{\upbeta\:}}}_{\mathrm{c}\mathrm{o}\mathrm{h}\mathrm{a}\mathrm{b}})$	$\:{\widehat{{\upbeta\:}}}_{\mathrm{c}\mathrm{o}\mathrm{h}\mathrm{a}\mathrm{b}}$	se($\:{\widehat{{\upbeta\:}}}_{\mathrm{c}\mathrm{o}\mathrm{h}\mathrm{a}\mathrm{b}})$	$\:{\widehat{{\upbeta\:}}}_{\mathrm{c}\mathrm{o}\mathrm{h}\mathrm{a}\mathrm{b}}$	se($\:{\widehat{{\upbeta\:}}}_{\mathrm{c}\mathrm{o}\mathrm{h}\mathrm{a}\mathrm{b}})$
2021	0.5	0	0.48	0.00	0.50	0.00	0.50	0.00	0.50	0.01
2020	0.5	0	0.49	0.00	0.50	0.00	0.50	0.00	0.50	0.00
2019	0.5	0	0.49	0.00	0.50	0.00	0.50	0.00	0.50	0.01
2018	0.5	0	0.48	0.00	0.50	0.00	0.50	0.00	0.50	0.01
2017	0.5	0	0.48	0.00	0.50	0.00	0.50	0.00	0.49	0.01
2016	0.5	0	0.47	0.00	0.50	0.00	0.51	0.01	0.49	0.01
2015	0.5	0	0.47	0.00	0.51	0.00	0.50	0.01	0.48	0.01
2014	0.5	0	0.46	0.00	0.50	0.00	0.50	0.01	0.49	0.01
2013	0.5	0	0.45	0.00	0.50	0.00	0.50	0.01	0.49	0.01
2012	0.5	0	0.45	0.00	0.51	0.01	0.50	0.01	0.48	0.01
2011	0.5	0	0.44	0.00	0.51	0.01	0.52	0.01	0.48	0.01
2010	0.5	0	0.43	0.00	0.51	0.01	0.52	0.01	0.46	0.01
2009	0.5	0	0.43	0.00	0.51	0.01	0.51	0.01	0.52	0.02
2008	0.5	0	0.43	0.00	0.51	0.01	0.50	0.01	0.47	0.01
2007	0.5	0	0.41	0.00	0.52	0.01	0.51	0.01	0.54	0.03
2006	0.5	0	0.40	0.00	0.52	0.01	0.52	0.01	0.53	0.03
2005	0.5	0	0.41	0.00	0.52	0.01	0.52	0.02	0.47	0.02
2004	0.5	0	0.40	0.01	0.52	0.01	0.50	0.01	0.49	0.03
2003	0.5	0	0.39	0.01	0.53	0.01	0.48	0.01	0.47	0.03
2002	0.5	0	0.38	0.01	0.52	0.01	0.51	0.02	0.52	0.04
2001	0.5	0	0.39	0.01	0.53	0.01	0.50	0.02	0.53	0.04
2000	0.5	0	0.37	0.01	0.52	0.01	0.51	0.02	0.53	0.05

Validation percentage indicates share of observations where true value is available to use for calibration. Validation exercises carried out using “mecor” package in R

## Conclusion

This study presented a way to assess the quality of recalled information in survey-based research by means of comparing against high-quality administrative records. Using cohabitation histories defined as spells of living with a partner (married or unmarried) as an example, the results contribute new knowledge of how recall error evolves over time. By comparing cohabitation histories collected in the Danish GGS program in 2021 with CPR-records, we were able to evaluate to what extent and under which conditions error increases when respondents are asked to recall cohabitation histories. Under mild assumptions, we quantify that quality of recall decreases by 0.6 (0.5) percentage point per year on average over a 20-year period, equivalent to an average of a 0.6% (0.5%) increase in recall error per year going further back in time.

### Limitations

The present study relies on a unique situation where the collected survey data could be compared to a ‘gold standard’ administrative data set. While this provides an opportunity for key insights into recall decay in cohabitation data, it does come with limitations. First, as also demonstrated in this study, administrative data comes with its own biases. Beyond individuals misregistered in the administrative data because they have failed to report a move, the main sources of bias likely come from the cohabitation definition. Living arrangements outside the normative definition of a relationship used in administrative records show up as measurement error, meaning that both co-living people, unmarried and childless cohabiting couples with additional roommates, people in nonstandard cohabiting relationships that include multiple partners, and unmarried and childless same-sex couples all will be misregistered in administrative data. However, as seen from Table 2, misregistered actual relationships for the reasons mentioned likely, at maximum, make up 1.7% of the 18–49-year-old population, whereas co-living individuals misregistered as couples at maximum make up 1.5%.

Second, such ‘gold standard’ data to compare full samples against are often unavailable to researchers, which is the very reason why survey data such as the GGS is invaluable. However, as shown, data need not be available for the full sample for calibration methods to be able to recover less biased estimates. Further, the size of validation set needed for calibrating estimates does not increase with sample size (Fox et al., 2020), so while obtaining such validation data can present a burden, it is not necessarily unsurmountable. For the present study of a little more than 7000 observations, as little as 5% of that sample (around 350 validation observations) proved sufficient, and this amount would not scale with sample size. Below we discuss several strategies where a small subset of data better validated may be available.

### Implications

In previous studies, the solutions to counter recall error necessitate either high-quality data or strong assumptions about the errors. In the worked example presented here only two mild assumptions were imposed, namely that of non-differential measurement error and that error only was present in one variable. In the presence of uncertainty about the nature of measurement error, the presented method can be adapted and used as a sensitivity test to gauge the potential effects of recall error under various scenarios. During data collection efforts, for example, researchers can ensure more thorough validation of key recall measures by collecting data from other sources for a subset of respondents and use this information to calibrate the recall error affected variable. This could include manual cross-validation against existing offline or non-link materials such as marriage records, tax records looking for joint filing status, or social security records. Similarly, triangulation through either re-interview or partner interviews (see Auriat, 1991) might also be an option for a subset of respondents. Alternatively, using more extensive modes of collection for a subset of respondents, such as event history calendars (Belli, 1998), could also be employed as a strategy to obtain more accurate information to calibrate against. Importantly, the findings from the present study indicate that obtaining such cross-validated information from even a small subset of the data material may allow for substantial bias correction.

The findings from this study can inform researchers of the magnitude and nature of potential bias when using recall instruments in surveys in general and in particular regarding cohabitation. Finally, a guided example for how to adjust for recall error using the *mecor*-package in R, if a small, random subset of observations includes true values exist, was provided. Such adjustment can likely prove invaluable in the assessment of data collected by means of recall instruments.

## Supplementary Information

Below is the link to the electronic supplementary material.

Supplementary Material 1

Supplementary Material 2

Supplementary Material 3

Supplementary Material 4

Supplementary Material 5

Supplementary Material 6

Supplementary Material 7

## Figures and Tables

**Fig. 2:**
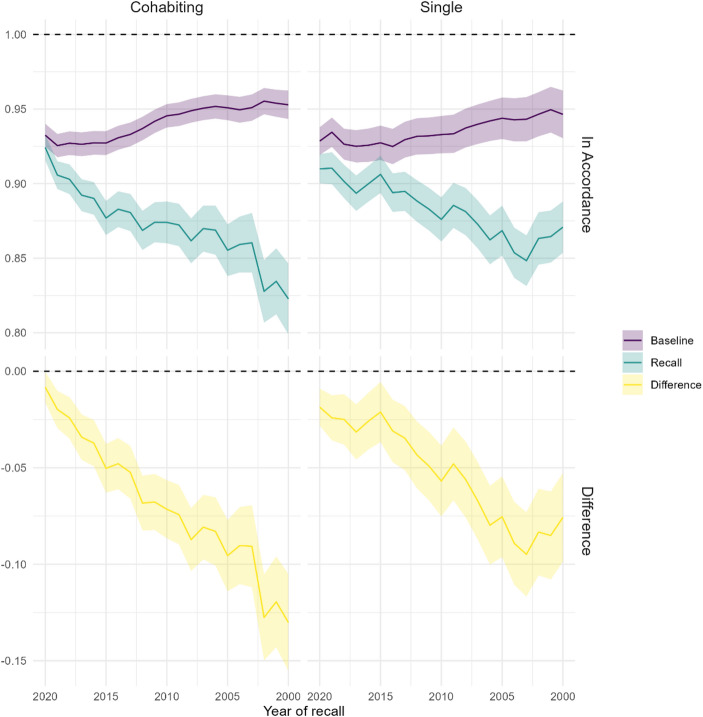
Reproducing Fig. 1a with unweighted data. Source: GGS DK 2020 and own calculations on data from Statistics Denmark.

## Data Availability

The present study received approval from Statistics Denmark under the auspices of data project no. 708085. The data underlying this article cannot be shared publicly due to privacy concerns restricting availability of register data for research. The author can make aggregated data available, conditional on ethical vetting. The author accessed the individual-level data through Statistics Denmark online access system. If a researcher at a university or other research institution outside Denmark wishes to use these data, this may be accomplished by visiting a Danish research institution or by cooperating with researchers or research assistants working in Denmark. All scripts to generate data and results underlying the study are available as Supplementary Materials.

## Appendix

See Fig. 2.

## References

[CR1] Andersson, G., Thomson, E., & Duntava, A. (2017). Life-table representations of family dynamics in the 21st century. *Demographic Research,**37*, 1081–1230. 10.4054/DemRes.2017.37.35

[CR2] Auriat, N. (1991). Who forgets? An analysis of memory effects in a retrospective survey on migration history. *European Journal of Population,**7*(4), 311–342. 10.1007/BF01796872 12284885 10.1007/BF01796872

[CR3] Ayhan, H. Ö., & Işiksal, S. (2004). Memory recall errors in retrospective surveys: A reverse record check study. *Quality and Quantity,**38*(5), 475–493. 10.1007/s11135-005-2643-7

[CR4] Beckett, M., Da Vanzo, J., Sastry, N., Panis, C., & Peterson, C. (2001). The quality of retrospective data: An examination of long-term recall in a developing country. *The Journal of Human Resources,**36*(3), 593–625. 10.2307/3069631

[CR5] Belli, R. F. (1998). The structure of autobiographical memory and the event history calendar: Potential improvements in the quality of retrospective reports in surveys. *Memory,**6*(4), 383–406. 10.1080/741942610 9829098 10.1080/741942610

[CR6] Berney, L. R., & Blane, D. B. (1997). Collecting retrospective data: Accuracy of recall after 50 years judged against historical records. *Social Science & Medicine,**45*(10), 1519–1525. 10.1016/S0277-9536(97)00088-9 9351141 10.1016/s0277-9536(97)00088-9

[CR7] Bingley, P., & Martinello, A. (2017). Measurement error in income and schooling and the bias of linear estimators. *Journal of Labor Economics,**35*(4), 1117–1148. 10.1086/692539

[CR8] Bound, J., Brown, C., & Mathiowetz, N. (2001). Chapter 59 measurement error in survey data. *Handbook of econometrics* (Vol. 5, pp. 3705–3843). Elsevier B.V. 10.1016/S1573-4412(01)05012-7

[CR9] Dalziel, K., Li, J., Scott, A., & Clarke, P. (2018). Accuracy of patient recall for self-reported doctor visits: Is shorter recall better? *Health Economics,**27*(11), 1684–1698. 10.1002/hec.3794 29968290 10.1002/hec.3794

[CR10] Fox, M. P., Lash, T. L., & Bodnar, L. M. (2020). Common misconceptions about validation studies. *International Journal of Epidemiology,**49*(4), 1392–1396. 10.1093/ije/dyaa090 32617564 10.1093/ije/dyaa090 PMC7750925

[CR11] Francesconi, M. (2005). An evaluation of the childhood family structure measures from the sixth wave of the British Household Panel Survey. *Journal of the Royal Statistical Society: Series A (Statistics in Society),**168*(3), 539–566. 10.1111/j.1467-985X.2005.00362.x

[CR12] Gertler, M., Czogiel, I., Stark, K., & Wilking, H. (2017). Assessment of recall error in self-reported food consumption histories among adults—Particularly delay of interviews decrease completeness of food histories—Germany, 2013. *PLOS One,**12*(6), Article e0179121. 10.1371/JOURNAL.PONE.0179121 28640839 10.1371/journal.pone.0179121 PMC5480875

[CR13] Gibson, J., & Kim, B. (2007). Measurement error in recall surveys and the relationship between household size and food demand. *American Journal of Agricultural Economics,**89*(2), 473–489. 10.1111/J.1467-8276.2007.00978.X

[CR14] Gibson, J., & Kim, B. (2010). Non-classical measurement error in long-term retrospective recall surveys. *Oxford Bulletin of Economics and Statistics,**72*(5), 687–695. 10.1111/j.1468-0084.2010.00599.x

[CR15] Goldschmidt, D., Klosterhuber, W., & Schmieder, J. F. (2017). Identifying couples in administrative data. *Journal for Labour Market Research,**50*(1), 29–43. 10.1007/S12651-017-0218-4/TABLES/7

[CR16] Haas, S. A., Glymour, M. M., & Berkman, L. F. (2011). Childhood health and labor market inequality over the life course. *Journal of Health and Social Behavior,**52*(3), 298–313. 10.1177/0022146511410431 21896684 10.1177/0022146511410431

[CR17] Havari, E., & Mazzonna, F. (2015). Can we trust older people’s statements on their childhood circumstances? Evidence from SHARELIFE. *European Journal of Population,**31*(3), 233–257. 10.1007/s10680-014-9332-y

[CR18] Hayford, S. R., & Morgan, P. (2008). The quality of retrospective data on cohabitation. *Demography,**45*(1), 129–141. 10.1353/DEM.2008.0005 10.1353/dem.2008.0005 PMC2722926 18390295

[CR19] Jivraj, S., Goodman, A., Ploubidis, G. B., & de Oliveira, C. (2020). Testing comparability between retrospective life history data and prospective birth cohort study data. *The Journals of Gerontology: Series B,**75*(1), 207–217. 10.1093/geronb/gbx042 10.1093/geronb/gbx042 PMC6909437 28444303

[CR20] Johnson, R. C., & Schoeni, R. F. (2011). The influence of early-life events on human capital, health status, and labor market outcomes over the life course B.E. *Journal of Economic Analysis and Policy*. 10.2202/1935-1682.2521/HTML 10.2202/1935-1682.2521 PMC3569741 23412970

[CR21] Jürges, H. (2007). Unemployment, life satisfaction and retrospective error. *Journal of the Royal Statistical Society: Series A (Statistics in Society),**170*(1), 43–61. 10.1111/j.1467-985X.2006.00441.x

[CR22] Keogh, R. H., Shaw, P. A., Gustafson, P., Carroll, R. J., Deffner, V., Dodd, K. W., et al. (2020). STRATOS guidance document on measurement error and misclassification of variables in observational epidemiology: Part 1—basic theory and simple methods of adjustment. *Statistics in Medicine,**39*(16), 2197–2231. 32246539 10.1002/sim.8532 PMC7450672

[CR23] Kjellsson, G., Clarke, P., & Gerdtham, U. G. (2014). Forgetting to remember or remembering to forget: A study of the recall period length in health care survey questions. *Journal of Health Economics,**35*(1), 34–46. 10.1016/j.jhealeco.2014.01.007 24595066 10.1016/j.jhealeco.2014.01.007

[CR24] Kreyenfeld, M., & Bastin, S. (2013). *Blurred Memory, Deliberate Misreporting, or “True Tales”? How Different Survey Methods Affect Respondents’ Reports of Partnership Status at First Birth* (No. WP-2013-017). Max Planck Institute for Demographic Research. http://www.demogr.mpg.de

[CR25] Kreyenfeld, M., Hornung, A., & Kubisch, K. (2013). The German generations and gender survey: Some critical reflections on the validity of fertility histories. *Comparative Population Studies,**38*(1), 3–28. 10.4232/10.CPoS-2013-02en

[CR26] Macdermid, R. H. (1989). The recall of past partisanship: Feeble memories or frail concepts?*. *Canadian Journal of Political Science,**22*(2), 363–376. 10.1017/S0008423900001347

[CR27] Manzoni, A., Vermunt, J. K., Luijkx, R., & Muffels, R. (2010). Memory bias in retrospectively collected employment careers: a model-based approach to correct for measurement error. *Sociological Methodology,**40*(1), 39–73. 10.1111/J.1467-9531.2010.01230.X

[CR28] Nab, L., van Smeden, M., Keogh, R. H., & Groenwold, R. H. (2021). Mecor: An R package for measurement error correction in linear regression models with a continuous outcome. *Computer Methods and Programs in Biomedicine,**208*, Article 106238. 34311414 10.1016/j.cmpb.2021.106238

[CR29] Perelli-Harris, B., & Lyons-Amos, M. (2015). Changes in partnership patterns across the life course: An examination of 14 countries in Europe and the United States. *Demographic Research,**33*(1), 145–178. 10.4054/DemRes.2015.33.6

[CR30] Powers, E. A., Goudy, W. J., & Keith, P. M. (1978). Congruence between panel and recall data in longitudinal research. *Public Opinion Quarterly,**42*(3), 380–389.

[CR31] Reimer, M. (2004). *Collecting event history data about work careers retrospectively: Mistakes that occur and ways to prevent them*. Max-Planck-Institut for Bildungsforschung. http://www.mpib-berlin.mpg.de

[CR32] Ruckdeschel, K., Sauer, L., & Naderi, R. (2016). Reliability of retrospective event histories within the German Generations and Gender Survey: The role of interviewer and survey design factors. *Demographic Research,**34*(1), 321–358. 10.4054/DemRes.2016.34.11

[CR33] Simonsen, E., Fallesen, P., Loft, L., & Mortensen, L. H. (2021). *The Danish generation and gender survey 2020: Data collection, access and quality of the data*. ROCKWOOL Foundation Technical Note

[CR34] Wollburg, P., Tiberti, M., & Zezza, A. (2021). Recall length and measurement error in agricultural surveys. *Food Policy,**100*, Article 102003. 10.1016/J.FOODPOL.2020.102003

